# Thermoplastic Phenomena and Morphological Changes upon Fast Pyrolysis of Biomass and Model Compounds

**DOI:** 10.3390/molecules30030700

**Published:** 2025-02-05

**Authors:** Francesca Cerciello, Christophe Allouis, Carmela Russo, Erik Freisewinkel, David Tarlinski, Barbara Apicella, Martin Schiemann, Viktor Scherer, Osvalda Senneca

**Affiliations:** 1 Istituto di Scienze e Tecnologia per l’Energia e la Mobilità Sostenibili (STEMS)-CNR, 80125 Napoli, Italy; francesca.cerciello@cnr.it (F.C.); christophe.allouis@cnr.it (C.A.); carmela.russo@cnr.it (C.R.); barbara.apicella@cnr.it (B.A.); 2 Institute of Energy Plant Technology, Ruhr University Bochum, 44801 Bochum, Germany; freisewinkel@leat.ruhr-uni-bochum.de (E.F.); tarlinski@leat.ruhr-uni-bochum.de (D.T.); schiemann@leat.ruhr-uni-bochum.de (M.S.); scherer@leat.ruhr-uni-bochum.de (V.S.)

**Keywords:** biomass, pyrolysis, softening, morphology, thermoplastic, spherodization

## Abstract

The work reports preliminary results on the morphological changes that biomass particles experience at high heating rates in a heated strip reactor (HSR) at T = 1000–1600 °C under an inert atmosphere. Samples included a natural lignocellulosic biomass (pinewood) as well as biomass components: cellulose, hemicellulose (xylan) and lignin. On top of that, reference compounds have been investigated, namely naphthalene pitch, a paraffinic wax and glucose. During the heat-up phase, the investigated biomass mainly retains the original morphology and size, while the single components exhibit different behaviors. Hemicellulose undergoes a fluid stage and eventually forms millimetric spherical char particles. Cellulose does not become fully fluid but softens and forms millimetric char aggregates of different shapes. Lignin particles hardly soften and stick together in a curved slab. Comparison with model compounds allows us to infer that the degree of melting and the viscosity of the melt are responsible for the final particle shape. In fact, naphthalene pitch and glucose appear to be more viscous during pyrolysis and lead to the formation of three-dimensional columns a few millimeters high. Wax undergoes extensive melting, but the relatively low viscosity and the absence of crosslinking reactions eventually lead only to the formation of droplets.

## 1. Introduction

During the heating and pyrolysis process in a boiler, carbon-based fuels such as coals, biomass and wastes undergo important structural reorganization, in parallel with volatile matter release. The biomass char particles produced at the end of pyrolysis may be spherical or irregular in shape, shrunken or swollen, and more or less porous. The shape and porosity of char particles have important implications on transport phenomena and fluid dynamics in boilers. These features contribute to the burning mode, ultimately impacting on conversion rate and efficiency. The relevance of these features is indeed acknowledged in coal combustion CFD (computational fluid dynamics) codes, which require particle size, swelling factor and porosity as input parameters. Several groups investigated the shape changes in coal [1] and biomass [2,3,4,5,6,7,8] particles in high-heating-rate (dT/dt > 10^3^ °C/s) devices. For brevity, just a few are cited here. Gil et al. [1] documented using SEM analysis that during fast pyrolysis in oxy-fuel atmospheres, coal particles soften and generate nearly spherical chars. The tendency towards spherodization at very high heating rates, which are typical of PF (pulverized fuel-fired) boilers, has also been reported for several biomasses; however, this is not a generalized phenomenon, and very different behaviors can also be found. Cetin et al. [2] investigated pine, eucalyptus and sugar cane bagasse in a drop tube and reported that bagasse was less prone to softening than the two wood species. Dell’Ora et al. [3] reported a more pronounced tendency towards spherodization for softwood compared to hardwood. McNamee et al. [5] investigated the influence of torrefaction on spherodization, reporting an increased tendency towards spherodization for torrefied eucalyptus and willow compared to untreated samples [5]. Panahi et al. [6] used char sampling (optical microscopy and SEM) and high-resolution videography to monitor the progressive shape changes of single biomass particles throughout pyrolysis in a drop tube. They observed a high tendency toward spherodization for miscanthus and a less pronounced tendency for beech. Panahi et al., in a further work [7], reported spherodization for five torrefied biomasses (corn straw, willow, pine, eucalyptus and miscanthus), even though only two spherodized as raw fuels as well (eucalyptus and miscanthus) [7], while Holmgren et al. [8] found that wheat biomass retained its original flake shape.

The physical and chemical processes that determine the shape and morphology of char particles have been addressed to some extent for coals in the past [9], but they are not completely understood. The reason for the observed differences among different biomasses is even more intriguing.

It is common sense that the swelling of solid fuels upon heat-up and pyrolysis would be related to the plasticity of the pyrolysis products, especially during the stage of incipient char formation, when the solidification of the carbon structure and the release of volatiles occur in parallel and in competition with each other. According to network pyrolysis models, like the chemical percolation devolatilization (CPD) model [9], after the labile bridges are broken, the fragments form the so-called “metaplast”. Part of the metaplast is released as volatile matter, while part of it reattaches to the solid matrix via crosslinking reactions. The fluidity of the metaplast and the rate of crosslinking could likely play a role in the formation of spherical vs. irregular char particles as well as on the formation of more graphitic vs. amorphous carbon. It could be expected that for biomass, the presence of different components (cellulose, hemicellulose, lignin, etc.) may contribute to the complexity of observed behavior.

The existence of an intermediate liquid phase during biomass pyrolysis has been debated in the literature for several decades [10,11,12,13,14,15,16,17,18,19,20,21,22]. In 1974, Nordin et al. [12] noticed that cellulose and biomass pass through a condensed phase during flash pyrolysis. It has been explained that during the pyrolysis of biomass, the lignocellulosic components depolymerize and the heavy oligomeric compounds with boiling points higher than the temperatures of the depolymerization reactions form a liquid intermediate phase [10,11,12,13,14,15,16,17,18,19,20,21,23]. Real-time visualization studies have shown that these heavy components in the liquid phase can be ejected in the form of aerosols through bubble explosions [13,14,15,16,17,18,19,20]. This phenomenon explains the presence of oligomeric products in bio-oil, which are typically not well characterized [22]. Dauenhauer et al. [13] recorded with a high-speed camera the evolution of cellulose during fast pyrolysis at 700 °C. Bubbling and evaporation of the melted phase promoted aerosol ejection and the shrinking of the liquid phase, without a trace of char formation. In 2011, Teixeira et al. [14] used a high-speed camera to visualize the mechanism of aerosol formation by bubbles bursting in the intermediate liquid phase during the pyrolysis of cellulose and sucrose; a new mechanism called reactive boiling ejection was proposed. Zhou et al. [20], using a high-speed camera, showed that, during lignin pyrolysis, lignin first melts. Then, bubbles are formed within the liquid phase, leading to particle swelling. Finally, evaporation and bubble collapse lead to particle shrinking.

Montoya et al. [21] examined the intermediate liquid phase formed during fast pyrolysis of biomass and of biomass components (cellulose, organo-solv lignin, milled wood lignin, xylan, de-ashed xylan, sugarcane bagasse and de-ashed sugarcane bagasse) by high-speed visualization techniques. The purpose was to identify the fraction responsible for the conservation of particle morphology. They confirmed that cellulose, lignin and de-ashed xylan were completely transformed into an intermediate liquid phase. Xylan, as received, did not fully melt and maintained its general shape during pyrolysis. These results suggest that the presence of mineral matter has a critical importance in allowing lignocellulosic materials to retain their original morphology.

Interesting clues about the thermoplastic behavior of biomass pyrolysis can be found in Dufour et al. [23], who examined the behavior of the main lignocellulosic components, namely cellulose, hemicellulose (xylan) and lignin. However, their study is limited to a relatively low temperature (about 450 °C) and heating rate (5 °C/min). The thermoplastic behavior of biomass and its constituents at very high heating rates, which are typical of fast pyrolysis systems (e.g., fluidized beds and pulverized firing boilers), requires further investigation, given the significance of such phenomena in defining particle morphology and shape, and ultimately, combustion performance in industrial burners.

The present work aims to contribute to understanding how the intrinsic properties of biomass and model compounds influence their structural transformations during pyrolysis. This knowledge is crucial for optimizing biomass processing to develop high-performance, sustainable pyrolysis and combustion processes. These issues may also be of interest for developing or enhancing lignocellulosic fibers and advanced materials, as they provide insights into biomass processing under extreme thermal conditions.

In this framework, the present work reports the results of an experimental campaign aimed at characterizing the melting phenomena and morphological changes in carbonaceous materials upon fast pyrolysis in a heated strip reactor (HSR), with heating rates up to 10^4^ °C/s and temperatures of 1000–1600 °C. The investigated samples include natural lignocellulosic biomasses, as well as isolated components, namely cellulose, hemicellulose (xylan) and lignin. The study is complemented by the investigation of selected reference compounds with different chemical compositions: naphthalene pitch, glucose and wax. A comparison with model compounds suggests that the degree of melting and the viscosity of the melt are responsible for the final particle shape.

## 2. Results

### 2.1. Biomass

**Figure 1:**
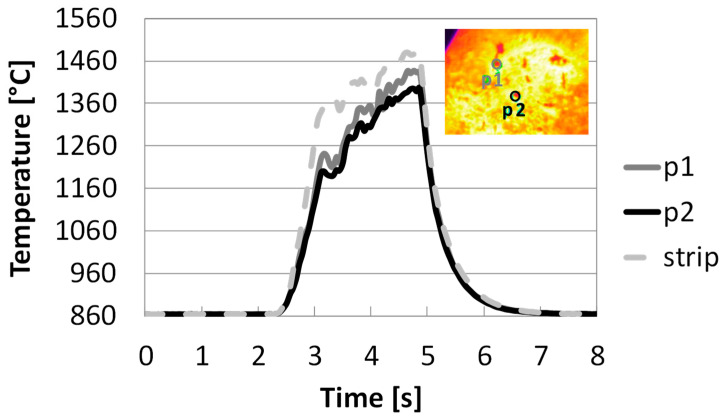
Temperature profile depending on the time of the reactor strip and two selected spots during pyrolysis in HSR at a target strip temperature ≈ 1500 °C (top view) (P1: dark grey solid line; P2: black solid line; Strip: dotted grey line).

Sequences of images taken by a thermocamera, observing particle samples on top of the heated strip, are presented and commented on in the following. Figure 1 reports the temperature profile depending on the time of two selected spots (p1 and p2) of particles with diameters of 0.01 cm during the pinewood pyrolysis at target strip temperature ≈ 1500 °C. The thermocamera is capable of registering frames from 630 °C (as detected with the pyrometer), even though it is calibrated from 860 °C. In all the experiments, particles are laid on the strip and care is taken to spread the sample uniformly, trying to avoid the formation of thick bulks of samples.

Figure 1 shows that reactor strip and particles are heated up simultaneously after t = 2.5 s. The strip temperature first rises rapidly in 0.5 s to 1360 °C, and then to 1480 °C after additional an 1.5 s. In the figure, 860 °C corresponds to the lowest value of the calibration temperature of the thermocamera. After the power is turned off, the strip and the particles cool down quickly in 2 s. Initially, the particles distributed on the strip can be assumed to be at the same temperature as the strip itself, while the development of agglomerates causes a temperature gradient between the strip and the top layers of the samples, which can surpass 100 °C.

**Figure 2:**
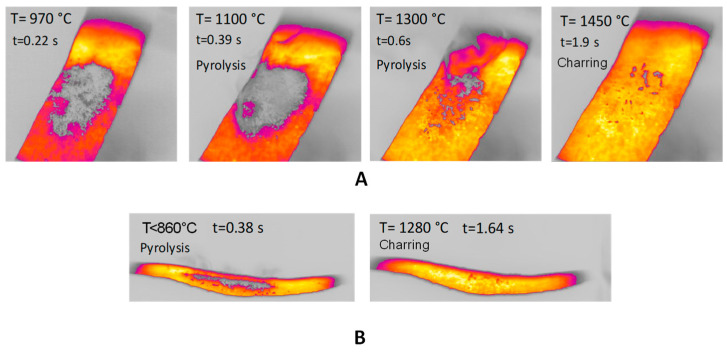
(**A**) HSR pinewood pyrolysis at a target strip temperature ≈ 1500 °C (top view); (**B**) HSR pinewood pyrolysis at a target strip temperature ≈ 1300 °C (lateral view).

Figure 2A reports the images obtained during experiments in the HSR of pinewood pyrolysis at a target strip temperature ≈ 1500 °C. The sample at the beginning of the experiments is seen as a layer of powder. Volatile flux is visible as a gray smoke in the figures, starting at t = 0.39 s and ending at t = 0.60 s. Time t_0_ is chosen as strip temperature reaches 630 °C. At the end of the experiment, a few dispersed particles are recovered from the strip. Figure 2B reports lateral views of pyrolysis in the HSR of pinewood at a target strip temperature ≈ 1300 °C. Although pyrolysis increases in this case, the underlaying phenomena remain similar. At the end of the experiment, the sample takes the form of fine char powder.

### 2.2. Biomass Components

**Figure 3:**
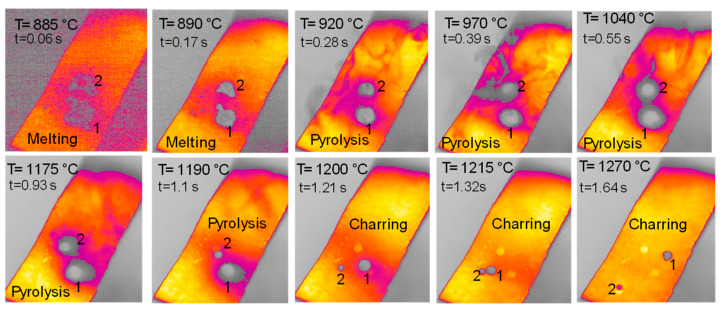
HSR experiment of hemicellulose at a target strip temperature ≈ 1300 °C (top view) [24].

**Figure 4:**
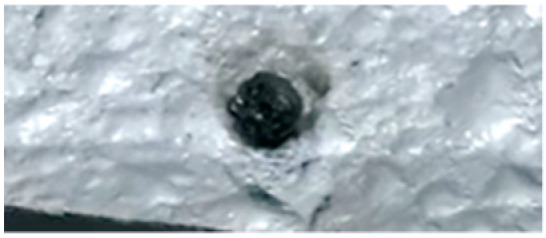
Char particle of hemicellulose collected in the HSR at a target strip temperature ≈ 1300 °C [24].

**Figure 5:**
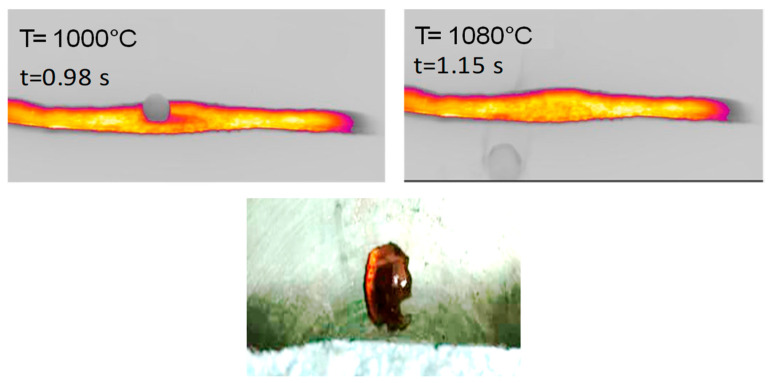
(**Top**) HSR experiment of hemicellulose at a target strip temperature of ≈1000 °C (lateral view). (**Bottom**) Brownish particle recovered at the end of the experiment [24].

**Figure 6:**
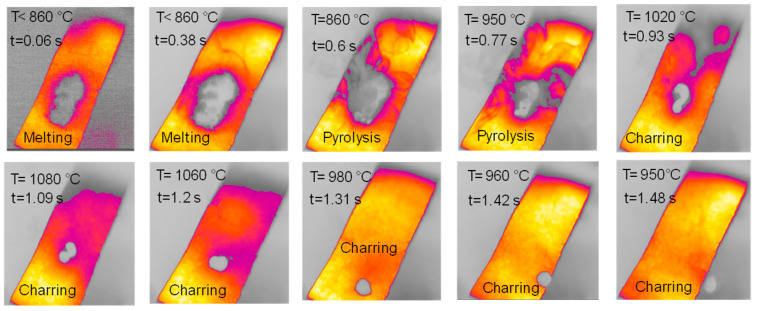
HSR experiment of cellulose at a target strip temperature ≈ 1000 °C (top view).

**Figure 7:**
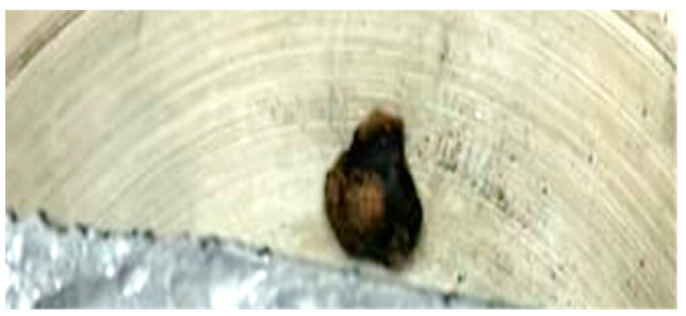
Particle recovered at the end of the cellulose pyrolysis experiment at a target strip temperature of ≈1000 °C.

**Figure 8:**
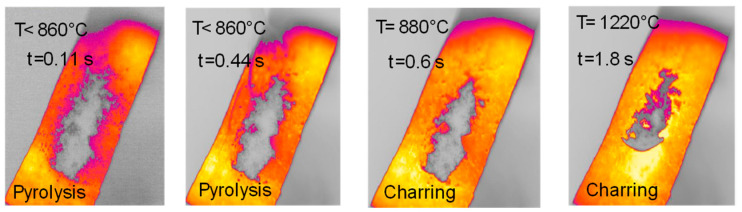
HSR experiment of lignin at a target strip temperature ≈ 1300 °C (top view).

Figure 3, Figure 4, Figure 5, Figure 6, Figure 7 and Figure 8 report images captured during pyrolysis in the HSR of the three lignocellulosic components investigated at a target strip temperature in the range of 1000–1300 °C: hemicellulose, cellulose and lignin.

The behavior of hemicellulose in the HSR is documented in the top view, shown in Figure 3, and the lateral view, shown in Figure 5, of the thermocamera during two similar pyrolysis experiments. Note that experiments on hemicellulose have also been reported in [24]. The sample of hemicellulose has an original cubical particle shape. In the experiment shown in Figure 3, two balks of particles of 1 × 0.8 cm and 0.8 × 0.6 cm are initially deposited on the strip. Between 0.06 s and 0.17 s, they generate two almost spherical agglomerates with a diameter of 0.6 and 0.5 cm and smoothed edges. Pyrolysis occurs when the strip’s temperature range is 900–1200 °C, and an intense release of volatiles forms a cloud of hot gas around the two clusters. As previously discussed, with larger agglomerates, the temperature gradient between the strip and the upper layers of the agglomerates is about 100 °C, but rolling phenomena can possibly lower these values. Shrinkage phenomena are observed at the end of the pyrolysis stage. Between 1.21 and 1.64 s, the charring stage takes place with a progressive shrinkage of the particle. Figure 4 reports the char particle (first spot) collected at the end of the experiment. Time, temperature and observed particle sizes of the two clusters during the experiments at a target strip temperature ≈ 1300 °C are reported in Table 1.

Lateral views of hemicellulose at strip temperatures of around 1000 °C are reported in Figure 5. These clearly show the formation of a spherical intermediate stage resembling molten droplets. At t = 1.15 s, the sphere moves along the strip and eventually falls off. A brownish particle is found on the reactor floor.

The sample of cellulose has an original spherical particle shape. The results for cellulose, documented in Figure 6, show that during heat-up a more complex and interesting behavior is found; a marked release of volatiles occurs between t = 0.38 s and t = 0.77 s, when the temperature reached 940 °C, and is accompanied by a progressive agglomeration of the particles lying on the strip. At approximately t = 1.2 s, a spherical cluster with a diameter of 0.5 cm forms, which migrates across the strip and eventually falls off. At the end of the experiment, a solid particle is recovered on the floor of the reactor, as shown in Figure 7. The recovered solid particle does not look like a char particle but more like an agglomeration of smaller sticky particles. Table 2 reports the time, temperature and observed particle sizes during the experiments in the HSR at a target strip temperature ≈ 1000 °C.

The sample of lignin has an original elongated particle shape. For lignin pyrolysis (Figure 8), volatile efflux is observed from t = 0.44 s to t = 0.60 s. At approximately t = 0.60 s, an agglomeration of sticky char particles is formed, which detaches itself from the strip, and eventually curves upwards. At the end of the experiment, the char sample appears as a single slightly curved slab (boat-shaped).

### 2.3. Model Compounds

**Figure 9:**
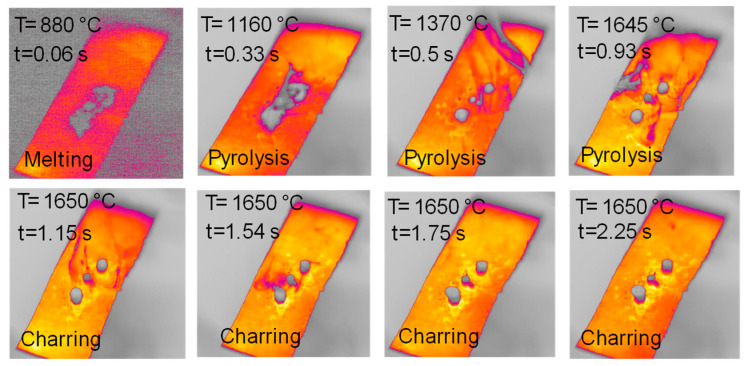
HSR experiment of naphthalene pitch pyrolysis at a target strip temperature ≈ 1600 °C (top view).

**Figure 10:**
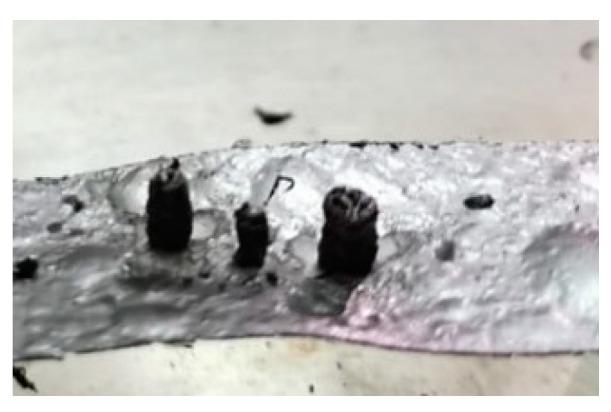
Particle recovered at the end of the naphthalene pitch pyrolysis experiment at a target strip temperature ≈ 1600 °C.

**Figure 11:**
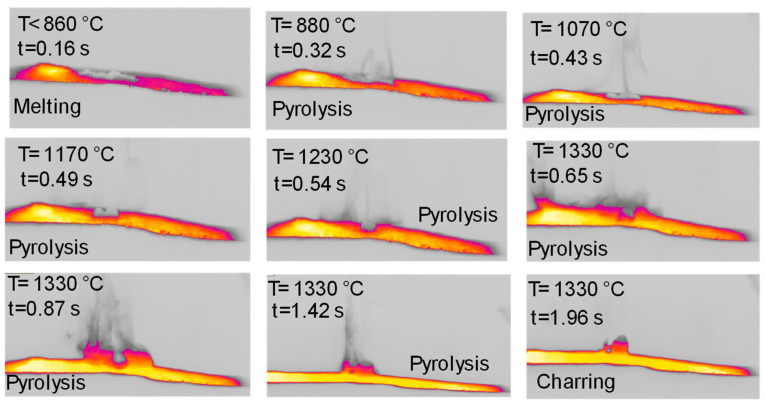
HSR experiment of naphthalene pitch pyrolysis at a target strip temperature ≈ 1300 °C (lateral view).

Figure 9 reports the top views of the pyrolysis experiments in the HSR on naphthalene pitch at a target temperature T ≃ 1600 °C. Notably, the behavior of naphthalene pitch is very interesting. In Figure 9, at t = 0.33 s, the powder dispersed on the strip undergoes pyrolysis, and the volatile efflux is quite strong, as also shown in the later photograms. At t = 0.50 s, the sample forms three distinct clusters on the strip. These clusters progressively grow in size in the vertical direction, forming three columns. At the end of the test, three porous black char columns of 7–10 mm in height are recovered on the strip (Figure 10). On the other hand, Figure 11 reports the lateral views of naphthalene pitch pyrolysis at a target temperature 1300 °C, with the evolution of volatiles and the formation of much shorter columns.

**Figure 12:**
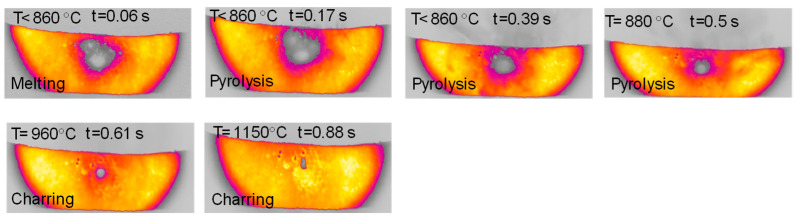
HSR experiment of glucose pyrolysis at a target strip temperature ≈ 1200 °C (top view).

**Figure 13:**
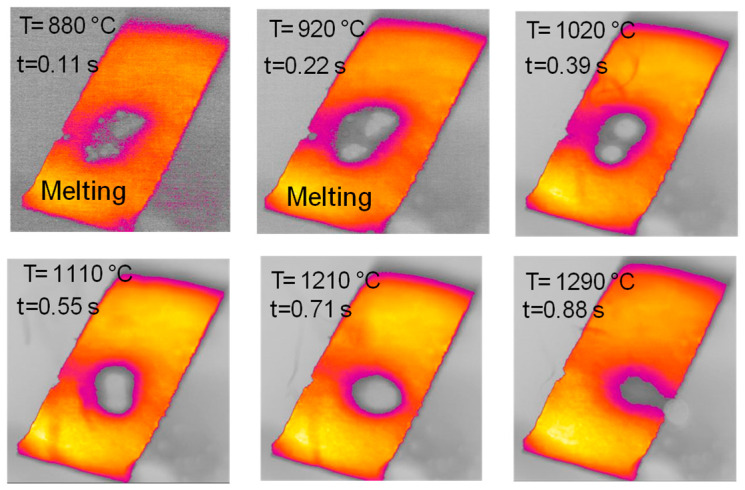
HSR experiment of wax pyrolysis at a target strip temperature ≈ 1300 °C (top view).

Figure 12 reports a sequence of images taken during an experiment with glucose in the HSR. In this case, the behavior is slightly similar to that observed for hemicellulose, with particles deposited unevenly on the strip forming multiple denser spots. Upon heat treatment, between t = 0.17 s and t = 0.39 s, an intense release of volatiles occurs. The multiple clusters of particles then merge on the strip to form a distinct sphere, which later progressively shrinks, ultimately forming one char column. Figure 13 reports a sequence of images taken during an experiment on wax in the HSR. Also in this case, the behavior is similar to that observed for hemicellulose, with an agglomeration of all particles into a spherical droplet, which moves on the strip and eventually falls off. The sample recovered on the floor of the reactor looks like a solidified wax drop.

## 3. Discussion

Wax, glucose and naphthalene pitch were chosen as representatives of aliphatic tars and “thermoplastic” organic compounds of biogenic (rich of oxygen) and fossil origin (mainly aromatics, without oxygen), respectively.

The most remarkable behavior upon pyrolysis in the HSR was found for naphthalene pitch, followed by glucose, with the formation of molten agglomerates which progressively grow in height. Wax, on the other hand, forms liquid droplets. This behavior could be attributed to the decreasing viscosity of the melted phase moving from naphthalene pitch over glucose to wax.

The particles of the investigated natural biomass, upon treatment on the heated strip, remained separate and did not show remarkable phenomena of melting and agglomeration. However, several examples of spherodization from lignocellulosic materials have been reported in the literature [2,3,4,5,6,7,8]. The investigated individual lignocellulosic components, however, showed melting and thermoplastic effects in the HSR.

**Figure 14:**
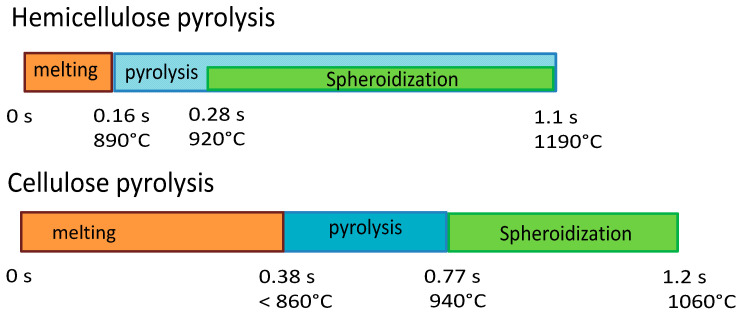
Timeline results from HSR experiments of hemicellulose at a target strip temperature ≈ 1300 °C and cellulose pyrolysis at a target strip temperature ≈ 1000 °C.

Figure 14 reports the timeline sequence of events registered during the experiments of cellulose and hemicellulose pyrolysis in the HSR. Hemicellulose reaches a clear fluid state, and in this form, it forms drops, similarly to wax. As pyrolysis proceeds, if the drop remains on the strip for enough time, it eventually forms a single spherical char particle. If the drop falls from the strip, it freezes into a glassy solid, with a shape similar to that produced by cooled wax. Cellulose has an intermediate behavior, forming a single large char particle with an imperfect spherical shape at the end of the devolatilization step. Lignin particles (not reported in Figure 14) experience more moderate melting, which leads to the particles sticking together in a slab.

Interesting clues about the thermoplastic behavior of biomass at a low heating rate (5 °C/min) and temperatures up to 400 °C have been provided by Dufour et al. [23], who performed an in situ study on the rheology and mobility of miscanthus, cellulose, lignin and hemicellulose throughout pyrolysis. At first analysis, the present HSR’s results and Dufour’s low temperature results are opposite; in the HSR, the biomass component with the highest propensity to pass through a fluid viscous state is hemicellulose, followed by cellulose and lignin, while in Dufour’s experiments, the opposite was found. However, these results align when the argumentation presented by Dufour et al. [23] is more carefully considered.

At the low heating rate and temperatures of [23], hemicellulose melted and softened from 200 °C. Dufour attributed the fluidity of this material to the mobility of primary pyrolysis products, since hemicellulose pyrolysis started at 200 °C. In the HSR experiments, because of the severe heating conditions, the pyrolysis rate is higher; thus, the fluid state of this material is reasonably favored in comparison to the mild heating conditions examined in [23].

As for lignin, in Dufour et al.’s work [23], it is reported that this component underwent glass transition before the start of pyrolysis (it was completely fluid at 200–225 °C). However, they also noted that lignin solidified as soon as pyrolysis and crosslinking reactions took off at around 350 °C. In the HSR, where these reactions are fast, it is therefore reasonable that the tendency of lignin to form fluid is hindered and counterbalanced by the tendency to form a crosslinked char.

Finally, for cellulose, Dufour et al. [23] reported that some increase in fluidity was observed at 250 °C, attributing it to the tendency of disordered carbohydrates to become liquid even before pyrolysis determines an appreciable mass loss. The formation of a more viscous melt compared to that of hemicellulose is also confirmed by the present experiments in the HSR, even though the progress of the pyrolysis reactions is, of course, more rapid under the heating conditions of the HSR.

The work of Dufour et al. [23] also highlighted potential interactions among the biomass constituents in natural biomass. For example, cellulose could impose its thermoplastic behavior on the overall structure and reduce the overall tendency toward melting. This could justify the inertia to melting and modest thermoplastic phenomena observed in the present work for the natural biomass investigated (pinewood). However, evidence of spherodization during the fast pyrolysis of other biomasses has been reported in the literature; therefore, a more extensive dataset of lignocellulosic biomass should be investigated in future works in order to elucidate whether and what interactions exist among lignocellulosic components in natural biomass of different origins and whether other constituents, such as extractives and minerals, could further interfere.

## 4. Materials and Methods

A selection of seven different samples is used for the experimental campaign:

1. pinewood—a typical woody biomass supplied by Luleå University of Technology (Luleå, Sweden) [25];
2. hemicellulose—xylan (from corncob) supplied by Carl Roth (CAS No. 9004-63-5, Karlsruhe, Germany) [26];
3. cellulose—vivapur MCC sphere supplied by JRS (CAS No. 9004-34-6, Rosenberg, Germany) [26];
4. lignin—dealkaline lignin supplied by Fisher Scientific (CAS No. 9005-53-2, Göteborg, Sweden) [26].
5. naphthalene pitch—a 100% mesophase pitch synthesized by polymerization of naphthalene, kindly provided by Mitsubishi Gas Chemical Company (CAS No. 25135-16-4, Tokyo, Japan) [27];
6. paraffinic wax;
7. D-(+)-glucose supplied by Sigma Aldrich (CAS No. 14431-43-7, Milano, Italy).

The samples have been seived to obtain particle sizes of 100–200 μm.

**Figure 15:**
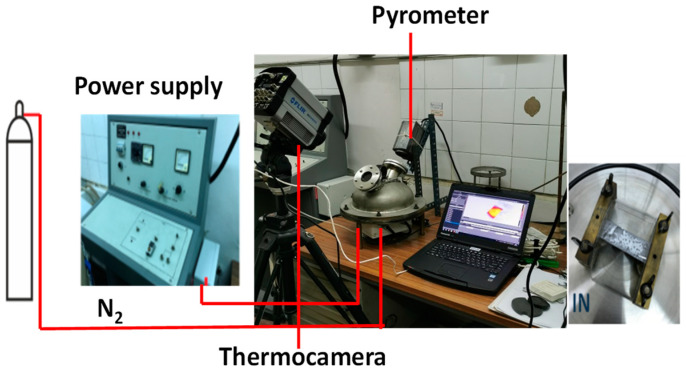
Heated strip reactor.

Figure 15 reports a photo of the equipment, the HSR and the equipment used in the experimental campaign. In the HSR, a pyrolytic graphite foil is used as the sample holder. The HSR is heated by Joule effects up to 2000 °C, a with heating rate of 10^4^ °C/s. The stainless steel reactor is equipped with two optical openings of quartz for a pyrometer or a thermal camera.

The HSR has been extensively used to investigate the fast pyrolysis of coal and biomass in nitrogen and carbon dioxide, with contextual analysis of the primary tars, by positioning a quartz bridge on top of the strip to collect the condensable products [26,28].

A fast FLIR thermal camera (FLIR SC6811), capable of capturing 38,000 frames, was used. The thermal camera can capture frames starting from 630 °C. Calibration was performed with the quartz window between the camera and the heated strip. Furthermore, a LAND R pyrometer, sensitive in the range of 630–2000 °C, was employed. Blank tests were conducted with various voltage and current settings to calibrate the thermal camera’s temperature readings against those of the pyrometer. In all the experiments, particles were laid on the strip and care was taken to spread the sample uniformly, trying to avoid the formation of thick bulks of samples. Our previous work [29] showed that the particles closely in contact with the strip can be considered to be the same temperature as the strip. Temperature differences between the bottom and upper surface of the particles were limited to 50 °C for particle sizes in the order of 100 μm, reaching 200 °C for larger particles. Once the sample was loaded, the reactor vessel was sealed and purged with nitrogen to eliminate any oxygen traces inside. The thermal camera captured the images and temperatures of the particles as they heated up. The heat treatments were varied between 1000 and 1600 °C, with a residence time set to 4–5 s.

After the power was turned off, the strip underwent rapid thermal cooling, the vessel was opened, and the residual solid sample (char) was recovered. The images captured by the thermal camera were processed to create time-temperature frames of the samples during heating, allowing for the tracking of particle morphology changes over time. Notably, in most of the experiments, the thermocamera acquired images from the top; however, some tests were repeated with a different positioning of the thermocamera in order to acquire lateral views.

## 5. Conclusions

The present work reports preliminary results on the morphological changes that solid particles of different types and origins experience upon very fast heating in an inert atmosphere. Samples included naphthalene pitch, paraffinic wax and natural lignocellulosic biomass (pinewood), as well as biomass components, namely cellulose, hemicellulose and lignin. The results, as summarized in Table 3, where the photos collected at the end of the pyrolysis experiments are reported, reveal distinct behaviors for the different materials:

- pinewood sample pyrolysis takes place without any relevant change in size or shape or evidence of melting;
- the lignin sample produces a curved slab of sticky powder char (boa-shaped) suggesting the formation of a highly viscous stage;
- cellulose forms an irregular spherical char, which moves on the strip and eventually falls down;
- hemicellulose forms a molten drop, which moves on the strip and eventually falls down. In cases where the melted drop remains on the strips, it turns into a spherical char particle.

Reference/model compounds have also been investigated:

- wax forms a molten drop, which moves on the strip and eventually falls down;
- naphthalene pitch forms three black porous char columns;
- glucose forms a char column.

These effects have been discussed and attributed to the melting and thermoplasticity of the materials upon high temperature pyrolysis, which certainly require in-depth analysis and theoretical interpretation in the follow-up of the research activity. The present investigation highlights evidence of an intermediate liquid phase in lignocellulosic components. The existence of the liquid phase within the particles has critical implications for defining mass and energy transfer during pyrolysis. Indeed, many physiochemical phenomena occur simultaneously within the liquid phase as solid/liquid reactions, mass and heat transfer in the liquid-solid-gas phases, and bubbling (nucleation, coalescence, growth). The HSR was chosen for its particular feature of reaching elevated temperatures with fast heating rates, heating up the sample in contact with the strip; however, it did not provide information on the change of viscosity with temperature.

## Figures and Tables

**Table 1:** Analysis of HSR experiment on hemicellulose at a target strip temperature of 1300 °C [24].

Stage	Time [s]	Temperature [°C]	Particles Dimensions [cm]	
			1st Spot	2nd Spot
	0 ^3^	630 ^3^	1 × 1 ^1^	0.8 × 0.8 ^1^
Melting	0.06	885	0.8 × 0.8 ^1^	0.8 × 0.6 ^1^
Melting	0.17	890	0.6 ^2^	0.5 ^2^
Pyrolysis	0.28	920	0.5 ^2^	0.4 ^2^
Pyrolysis	0.39	970	0.5 ^2^	0.4 ^2^
Pyrolysis	0.55	1040	0.5 ^2^	0.4 ^2^
Pyrolysis	0.93	1175	0.45 ^2^	0.35 ^2^
Pyrolysis	1.1	1190	0.4 ^2^	0.3 ^2^
Charring	1.21	1200	0.4 ^2^	0.2 ^2^
Charring	1.32	1215	0.3 ^2^	0.2 ^2^
Charring	1.64	1270	0.2 ^2^	0.1 ^2^

^1^ Length (L) × width (W); ^2^ diameter; ^3^ time t = 0 is taken as the time when the strip reaches 630 °C, which is the lower temperature limit of the pyrometer.

**Table 2:** Analysis of HSR experiment of cellulose at a target strip temperature of ≈1000 °C.

Stage	Time [s]	Temperature [°C]	Particles Dimensions [cm]
			1st Spot
	0 ^3^	630 ^3^	1 × 2 ^1^
Melting	0.06	<860	1 × 2 ^1^
Melting	0.38	<860	1 × 2 ^1^
Pyrolysis	0.6	860	0.7 × 2 ^1^
Pyrolysis	0.77	940	0.5 × 1 ^1^
Charring	1.09	1080	0.4 × 0.8 ^1^
Charring	1.2	1060	0.4 × 0.6 ^1^
Charring	1.31	980	0.5 ^2^
Charring	1.42	860	0.5 ^2^
Charring	1.48	860	0.5 ^2^

^1^ Length (L) × width (W); ^2^ diameter; ^3^ time t = 0 is taken as the time when the strip reaches 630 °C, which is the lower temperature limit of the pyrometer.

**Table 3:** Char morphologies.

Char powder: Biomass (pinewood)	** 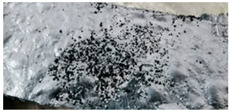 **
Char curved slab: Lignin	** 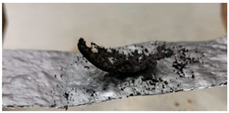 **
Porous char columns: Naphthalene pitch, glucose	** 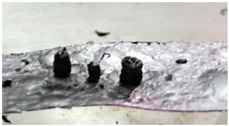 **
Molten drop: Hemicellulose, wax	** 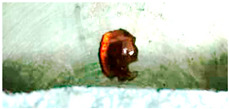 **
Spherical char: Hemicellulose	** 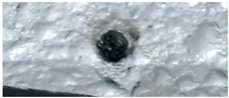 **
Irregular spherical char: Cellulose	** 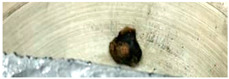 **

## Data Availability

The dataset is available from the authors upon request.
